# A Novel Anti-Environmental Forest Experience Scale to Predict Preferred Pleasantness Associated with Forest Environments

**DOI:** 10.3390/ijerph17186731

**Published:** 2020-09-16

**Authors:** Ernest Bielinis, Jianzhong Xu, Aneta Anna Omelan

**Affiliations:** 1 Department of Forestry and Forest Ecology, Faculty of Environmental Management and Agriculture, University of Warmia and Mazury in Olsztyn, Pl. Łódzki 2, 10-727 Olsztyn, Poland; 2 Department of Counseling, Educational Psychology, and Foundations, Mississippi State University, Starkville, MS 39759, USA; jx18@colled.msstate.edu; 3 Faculty of Education, University of Macau, Macau 999078, China; 4 Department of Tourism, Recreation and Ecology, Faculty of Environmental Sciences, University of Warmia and Mazury in Olsztyn, ul. Oczapowskiego 5, 10-719 Olsztyn, Poland; aneta.omelan@uwm.edu.pl

**Keywords:** forest environments, forest experience, psychometric test

## Abstract

In this study, a method for predicting the preferred pleasantness induced by different forest environments, represented by virtual photographs, was proposed and evaluated using a novel Anti-Environmental Forest Experience Scale psychometric test. The evaluation questionnaire contained twenty-one items divided into four different subscales. The factor structure was assessed in two separate samples collected online (sample 1: *n* = 254, sample 2: *n* = 280). The internal validity of the four subscales was confirmed using exploratory factor analysis. Discriminant validity was tested and confirmed using the Amoebic Self Scale (spatial–symbolic domain). Concurrent validity was confirmed using the Connectedness to Nature Scale. Predictive validity was based on an assessment of pleasantness induced by nine different photographs (control—urban landscapes, forest landscapes, dense forest landscapes), with subscales differently correlated with the level of pleasantness assessed for each photograph. This evaluation instrument is appropriate for predicting preferred pleasantness induced by different forest environments.

## 1. Introduction

### 1.1. Importance of a New Questionnaire

Experiencing the natural landscape in everyday life is an important factor associated with human wellbeing [1,2,3,4]. Contact with nature is also crucial for psychological health since there are many nature-based therapies, including horticulture therapy [5,6] and forest therapy [7,8,9], which are useful in treating depression and other diseases. The importance of forests, as a special type of natural environment, is also high. For example, in Poland, a medium-sized Central European country located in the range of temperate forest biomes, forests cover close to 29% of the landscape [10]. Much of this area is natural and free from significant human infrastructure. Forest areas have great accessibility and potential for use in forest therapy experiences, an outdoor recreational activity which might be used as a remedy for stress [11,12]. In Poland, this activity is used as therapy in different seasons [13,14]. Thus, Poland is a good example of a country with accessible forest resources for forest therapy. The forest landscape can also be represented by virtual natural environments, i.e., by viewing images of forest landscapes on electronic devices, when real natural experiences are not accessible. Viewing virtual forest environments also has a positive influence on psychological relaxation [15,16,17,18].

Nevertheless, because viewing forest landscapes is critical to reducing stress and obtaining an optimal psychological state, it is also important to know if subjects obtain similar psychological benefits from viewing a virtual representation of these environments. Methods for predicting the extent of this benefit are in an early stage of development [19]. Currently, there is no available questionnaire which can predict the level of psychological relaxation obtained by each subject. It is easy to imagine that, in the future, when physicians want to prescribe a treatment for a patient, they will be able to administer a questionnaire to see if a person will benefit from a specific treatment such as viewing a virtual or real forest. This situation will be possible if an instrument for predicting the psychological impacts of different forest experiences has been developed. The Anti-Environmental Forest Experience Scale (AEFES) questionnaire can also be used to predict the effects that different forms of activity may have during forest therapy. Looking at the forest, walking in the forest and touching various objects in the forest may be associated with different levels of experienced benefits from such an activity, which can be predicted by using the AEFES questionnaire in advance in patients participating in these activities.

### 1.2. Theoretical Framework for Building the Questionnaire

There are two promising theories for determining the fundamental psychological indices of an individual which might be usable for prediction of the level of psychological relaxation experienced in forest environments or in virtual reality. The first is Amoebic Self Theory [20], which describes the human self as similar to the construction of amoebas, in that the task of the amoeba is to assess differences between self and non-self (the physical domain of amoebic self), the difference between friend and foe (the social domain of amoebic self) and mine versus non-mine (the spatial–symbolic domain of amoebic self). The most important (from the point of view of our considerations) are the names of objects which might be seen, touched, smelled or heard in the forest. These kinds of objects might include trees, shrubs, plants, etc. in the forest, which might be seen as non-self objects. However, according to this theory, these objects might be involved in the self of the object on a different level; hypothetically, if the object is further away, then its influence on the boundaries of the amoebic self is lesser. So, according to these considerations, objects which touch the body, like insects, should generate more potential discomfort (as the boundaries of the self are more vulnerable to violation), while seeing herbivores, like deer, from a distance should generate a lower level of discomfort. Reactions to objects also have evolutionary backgrounds, because it is important to survive and to protect oneself from a potentially dangerous phenomenon, such as insects, which could spread a disease [21]. However, none of the domains of the Amoebic Self Scale exactly connect with phenomena that occur in forest environments. The closest is the spatial–symbolic domain, which involves categorizing objects as mine versus non-mine.

The other theory connected with the reactions of individuals in forest landscapes is the Prospects and Refuges Theory [22]. According to this theory, people feel safe when they have an open (prospect) view of the landscape and also feel safe if the landscape does not have places in which carnivorous or other dangerous animals can hide (no refuges). This reaction also has an evolutionary background because this is a mechanism for surviving in the dangerous natural environment. This theory suggests that only high-prospect and low-refuge landscapes will induce optimal psychological relaxation [23].

### 1.3. Anti-Environmental Forest Experience

Insofar as there is some possibility of predicting the level of psychological relaxation and therefore preferred pleasantness induced by viewing different forest landscapes, it is important to propose new concepts for these contexts. Biodiversity of urban green spaces is a predictor of their psychologically restorative benefits [24]. Other research has shown that amenities, incivilities and usability are predictors of park satisfaction [25]. Environment preference and environment type congruences may also be used to predict effects on perceived restoration potential and restoration outcomes [26]. It may also be possible to use the Natural Environment Scoring Tool to assess the impact of natural environments on psychological relaxation, but this tool was not applied in the forest and is not designed to test this environment [27]. There are few research tools for assessing how individual preferences predict the potential preferred pleasantness or other indices of psychological relaxation obtained by visual stimulation from forest landscapes, although life satisfaction tools may be used to predict the effect of forest environments on psychological relaxation [28]. Other personality indices, such as the big five personality tool or Myers-Briggs Type Indicator (MBTI) personality tools, may be successfully used as predictors of psychological satisfaction in the forest [29,30].

The Anti-Environmental Forest Experience approach proposed here may provide a way of integrating the Amoebic Self Theory and Prospect and Refuge Theory into a unified theory, something currently missing in the literature. The term “anti-environmental” for this new scale has been chosen in order to emphasize the fact that the attitude of those who are less likely to benefit from being in a forest environment is against (anti) features of that environment—features of the forest environment, such as the presence of plants, forest litter, animals and insects, are viewed as negative and potentially discomforting.

An Anti-Environmental Forest Experience proposes that the human self has an integrated psychological mechanism which works like the membrane of an amoeba, and this membrane divides the self from the environment. In this respect, it is similar to the Amoebic Self Theory, in which objects are separated from the self [20]. The Anti-Environmental Forest Experience has an evolutionary background similar to the Prospect and Refuge Theory, because being able to distance oneself from the environment might increase chances of survival, especially in dense forests, in which potentially dangerous animals such as carnivores, poisonous snakes or spiders might be hidden [22]. This boundary might also provide protection against potential pathogens in the environment. Things that provoke disgust or fear of violating the boundary of the self are aspects of the Anti-Environmental Forest Experience that work to protect the self from danger [21]. This study considered the possibility of contact in the temperate forest with different environmental interactions. These interactions were (1) contact with plants and litter, including contact with plants growing in the forest, such as touching trees, shrubs and other plants, or the smell of leaf litter; (2) seeing animals, particularly those which are skittish and usually visible only from a distance; (3) unpleasant situations such as coming across the carcass of a dead animal; (4) contact with insects and other signs of small animals, such as finding ticks or spider webs. One might also encounter other humans in the forest [31]—for example, human-made infrastructures such as huts, buildings, forestry machines, pylons—but this possibility was not considered in this study, because this is not an interaction with an object in the natural environment.

In these studies, based on the authors’ considerations and people’s relations to the forest, four factors were selected that may best reflect the interaction of the human body with the forest environment. During the construction of the AEFES questionnaire, a list of situations that may occur in the forest and that may potentially cause discomfort to participants of the walk in the forest was made; the conditions of the forest from the temperate zone were taken into account. Thus, in a temperate forest, the body may interact with the forest litter and plants, because people walking in the forest reported discomfort from the plants that touched them, and the smell and consistency of the forest litter, so a set of statements for factor 1 was created. Participants in forest walks reported their interaction with animals in the forest, which is an example of a situation where discomfort is caused by objects that are far away from the body but can potentially damage them in the interaction, so the items were selected to create factor 2. During the walk in the forest, participants also reported discomfort related to the feeling of disgust in the forest, which could cause discomfort with potential closer contact with the body; therefore, a set of statements for factor 3 was created.

Many people who took walks in the forest reported their discomfort in contact with insects in this environment and created a set of statements presenting the situation of contact with insects, forming factor 4. The proposed four factors may allow for a better understanding of the phenomenon of “discomfort” felt in the forest, because they explain psychometric scales measuring such traits as emotions better than previously used in research on psychological relaxation (Positive and Negative Affect Schedule), mood (Profile of Mood States, including the item “comfort”) or restorativeness (Perceived Restorativeness Scale) [12,13,14,28,29]. These scales only allow respondents to express discomfort experienced in the forest environment in general terms, but the proposed AEFES scale factors allow for a deeper understanding of this phenomenon, because the example “comfort” from the Profile of Mood State (POMS) scale captures this issue in one statement, and the AEFES scale breaks them down into twenty-one statements and four factors. This allows a deeper understanding of the issue of discomfort in the forest environment.

### 1.4. Aim of the Study, Plan of Analysis and Expected Outcome

Based on this consideration, it is probable that humans have self-boundaries vulnerable to violation in each of these four aspects. The Anti-Environmental Forest Experience Scale (AEFES) was created to test the hypothesis that human self-boundaries are vulnerable to violation by each of these four aspects. An explanatory factor analysis (EFA) was conducted on two samples, as were reliability, concurrence, discriminant and predictive validity tests. The EFA analysis was designed to test the extent to which the four proposed subscales of AEFES confirm the Anti-Environmental Forest Experience theory. The reliability of these four aspects should consider their viability as integrated constructs and their correlation with other scales. The reliability of these scales provides evidence that an Anti-Environmental Forest Experience can help predict the pleasantness induced by viewing forest landscapes and can be useful in predicting potential benefits that might be obtained by a subject from nature-based therapy. Preferred pleasantness can be combined with a feeling of violation of self-boundaries because this violation of self-boundaries is seen in this work as one of the determinants of preferred pleasantness—the more natural self-boundaries are sensitive to factors that can be supplied from outside (caused by viewing photos of landscapes), the less will be the perceived preferred enjoyment of these landscapes. High values obtained on the scale measuring the sensitivity of self-boundaries will be associated with the prediction of a low level of benefits that will be achieved by viewers of the forest landscape.

Certain expectations about concurrent validity were made. Among these was the expectation that the Amoebic Self Scale (spatial–symbolic domain, AmSS-SS) and AEFES should both measure vulnerability to violations of self-boundaries. Because, in social psychology, there are sometimes small correlations between constructs [20] and also because AmSS-SS measures similar, but not identical, things (AmSS-SS measures identification with self and non-self-objects, while AEFES measures hypothetical anti-environmental attitudes of the self rather than identification), the supposition was made that the correlation between these two scales should be significant, positive and with Pearson’s *r* values in the range of 0.2–0.4.

In a discriminant validity verification procedure, the scale should be inversely correlated with another construct, not measuring the same thing or measuring something opposite. The correlation between AEFES concurrent construct should be significantly negatively correlated with a moderate Pearson’s *r* correlation coefficient of more than 0.1 but not more 0.4. In this study, the Connectedness to Nature Scale (CNS) [32] was used as a discriminate validity scale. This scale measures emotional connectedness with the environment and relates to an individual’s attachment with the environment. Because place attachment, one of the primary constructs in environmental psychology, was correlated significantly with CNS at a level of *r* = 0.25 [33], it is assumed that CNS will be negatively correlated with AEFES as it measures the subject’s self and personal attachments. Since, theoretically, the pro-environmental construct should be CNS, a negative correlation should be obtained with anti-environmental AEFES, which measures anti-environmental forest experiences.

The predictive validity of the scale depends on its ability to predict values of importance to the researcher or practitioner. In this case, evaluating the AEFES scale depends on its ability to predict preferred pleasantness. Subscales of AEFES should be negatively correlated with the positively evaluated construct, such as the level of pleasantness which a subject obtains from viewing forest landscapes. It is also possible to see a positive correlation compared to control, urban landscape images. The forest landscape might be viewed by subjects as non-preferred by images, with high levels in AEFES; thus, a negative correlation might be observed. Because the urban landscape is not associated with plants, litter, animals and insects, no correlation or even a positive correlation should be observed with preferred pleasantness and AEFES. This study used only the photographic representation of a landscape for research; thus, the magnitude of the expected correlation is not high, yet it is still significant. It should be in a range between 0.1 and 0.4, though it is likely that it would be higher in the real, natural environment.

Looking at the virtual forest landscape may evoke various impressions, usually positive, which has been proven in research [15,16,17,18]. In the study by Korpela [19], it was shown that looking at photos of a forest landscape is associated with higher values of preferred pleasantness, which was correlated with perceived restorativeness (*r* = 0.72–0.87). Since looking at pictures of the forest evokes positive feelings (compared to pictures of an urbanized area), for this reason, it was decided in this experiment to use virtual pictures to produce an effect that could be predicted using the proposed AEFES scale.

## 2. Materials and Methods

### 2.1. Participants and Procedure

Two groups of participants participated in this study. The first group, designated as “Study 1”, had 254 participants. These participants were invited to participate via personal invitations extended by a forestry student at the University of Warmia and Mazury in Olsztyn on social media. Participants filled in a questionnaire on a specifically prepared website. The data of two respondents are not included in the study, because they did not give permission to use the data for research purposes. Data were collected between 22 January 2020 and 2 March 2020. The second group involved, “Study 2”, had 280 participants. These participants were also recruited via social media, with technical assistance from the Department of Forestry and Forest Ecology. Responses were collected between 4 February and 27 February 2020. The Forestry and Forest Ecology Department webpage was also used to advertise the questionnaire. All participants in Study 2 agreed to share their responses for this study. Participation in both study groups was voluntary. All participants were Polish nationals. In both study groups, half of the respondents had backgrounds in nature education or work, while the other half did not.

The online questionnaires were prepared on two special websites. The first questionnaire contained demographic questions about the participant, the AEFES, CNS and other scales that are not significant for this study. The second questionnaire contained questions about the demography of the participants, the AEFES, CNS, spatial–symbolic aspects of the Amoebic Self Scale and nine photographs used to evaluate pleasantness using a scale of 1–3. Each photograph was presented on a separate page, with the photographs displayed in random order. To avoid the bias of viewing the photographs of the landscape before filling the questionnaire, it was possible to see and evaluate each photograph on the pleasantness scale only after filling in the other scale.

Because the study involved human subjects, it was reviewed and approved by the Ethical Review Board at the University of Warmia and Mazury in Olsztyn. The number of the ethical statement is 06/2019. All procedures performed in this study were in accordance with the ethical standards of the Polish Committee of Ethics in Science and with the 1964 Helsinki Declaration’s later amendments.

### 2.2. Demographic Information

Participants in both study groups were asked to fill in demographic data on the questionnaire, including their gender, age, type of residence (ranging from a village to a big city of more than 250,000 inhabitants), educational background (from primary school to more than bachelor degree), self-evaluation of material standing (on a five-point Likert scale, from “1—very bad” to “5—very good”) and employment (working, unemployed or student). After filling in these data, participants proceeded to the other website for further questions (each visible on a separate page). The demographic information about each study group is shown in Table 1.

### 2.3. Instruments

#### 2.3.1. AEFES

Based on previous theoretical considerations, a questionnaire was designed to measure the level of discomfort experienced by a subject. Participants were asked to imagine that they were hypothetically involved in an exceptional situation. On a seven-point Likert scale from “1—strongly disagree” to “7—strongly agree”, the participants evaluated whether this exceptional situation induced discomfort. Four a priori groups were proposed: (1) contact with plants and leaf litter in the forest (Plants and litter), with participants asked about the level of discomfort induced by six specific situations, such as “head hit by a falling cone” or “body hit by a branch”; (2) encounters with animals in the forest (Animals), such as “seeing a deer with antlers from afar” or “seeing a fox from afar”; (3) seeing a disgusting thing in the forest (Disgust), with five encounters proposed, i.e., “seeing a slithering snake” or “finding a dead animal”; (4) contact with insects in the forest (Insects), with six encounters proposed, including “being bitten by a ‘horse fly’” and “being swarmed by insects”. It is worth mentioning that these items are connected with real discomforts which might feasibly happen in the forests of Central Europe. In other parts of the world, these encounters might be different, depending on the circumstances of the local environment.

Sensitivity to discomfort may measure the hypothetical boundaries of the self, which may vary for each participant. In this case, the involuntary violation of these boundaries by the forest environment was measured, because situations occurring in the forest are not wanted by each subject and subjects have individual reactions to the environment which are connected with their self-boundaries [20].

#### 2.3.2. Spatial–Symbolic Domain of Amoebic Self Scale

Burris and Rempel [20] proposed that the human’s amoebic self has several domains. One of these domains is the spatial–symbolic domain, which is connected with feelings of fear and disgust [34]. This domain describes the relationship between mine and non-mine objects and allows the recognition that different subjects have different degrees of vulnerability to these self-boundaries being violated. This vulnerability is possible to measure by the Amoebic Self Scale, in which the subject provides answers to a list of hypothetical involuntary situations that might occur in their life and influences their identity. There is a Polish adaptation (with some modifications) of these domains containing a list of 10 hypothetical situations which might induce discomfort. The reported Cronbach’s α was varied between 0.7 and 0.71 for these domains [34]. The discomfort induced by each situation was evaluated by subjects on a 7-point Likert scale (from “1—strongly disagree” to “7—strongly agree”). Examples of items used in this scale are “The thought of getting amnesia, of forgetting who I am, is really disturbing to me” and “I am disturbed when I think that there may be aliens or extraterrestrials who will someday invade Earth”. This domain has not previously been examined in the context of the temperate forest environment. The authors suppose that this domain is only slightly correlated with other aspects connected with the self: the Anti-Environmental Forest Experience. Admittedly, this scale measures some aspects of the vulnerability of self-boundaries but not other aspects connected with vulnerability to contact with the natural environment. As such, the authors suppose that only a slight correlation will occur, but such a correlation will be present. Thus, the correlation between these two scales will be used as a concurrent validity example in the current investigation.

#### 2.3.3. CNS

CNS is a psychometric tool which can be used to measure an individual’s emotional connection to the natural world [32]. This scale is reliable and valid, with an internal consistency of α = 0.72 [32]. The Polish adaptation of the scale is also available [35,36]. Participants responded on a 5-point Likert scale from 1 being “completely disagree” to 5 being “completely agree”. Example items for this measurement are “My personal welfare is independent of the welfare of the natural world” and “I think of the natural world as a community to which I belong”.

This scale is connected with experiences of people’s relationship to the natural world but not related seriously to the boundaries of the self. Thus, CNS is used in the current study as a measure to assess the discriminant validity of the main instrument.

#### 2.3.4. Photographs of Landscapes and Their Pleasantness Evaluation

Photographic representations of landscapes were used to assess the suitability of AEFES. For these purposes, nine photographs of three types of landscapes were prepared. Type 1 contained three photographs of an urban environment (views of three urban points near the campus of the University of Warmia and Mazury in Olsztyn were photographed for this purpose). Type 2 contained three photographs of forest landscapes (three different forest landscapes were photographed: the first in Central Park in Helsinki, the second and third near the campus in Olsztyn). Type 3 contained three photographs of a dense forest located near the campus. The use of these three groups was based on the results of a previous scientific study because, as was supposed, the study suggested that photographs from urban environments would induce low pleasantness, photographs of forests will induce high pleasantness, and photographs of a dense forest will produce high pleasantness (but lower than the forest landscape without disturbing factors like shrubs or undergrowth) [23,37].

**Figure 1:**
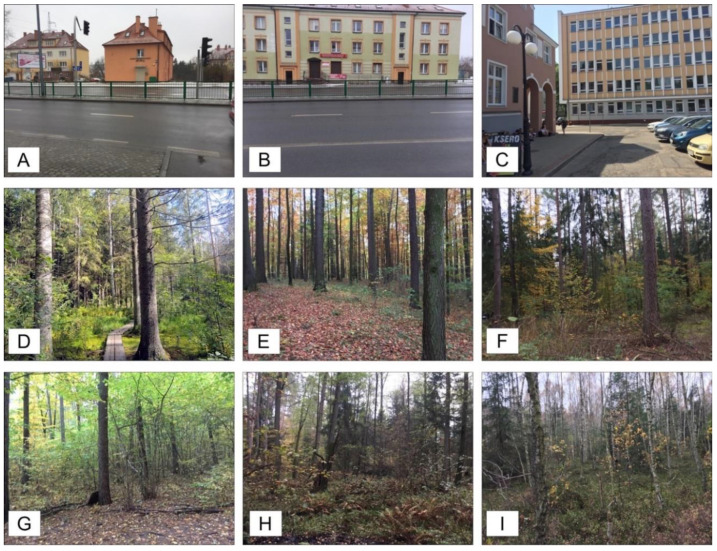
The photographs present three types of landscape: (**A**–**C**) present urban landscapes, (**D**–**F**) present forest landscapes and (**G**–**I**) present dense forest landscapes. Each photograph was used in this study for measuring preferred pleasantness.

For preparing all landscape photographs, a smartphone iPhone 6 was utilized. During landscape photographing, the operator was focused on representing the possible maximal natural experience of the viewer of the landscape by keeping the smartphone at eye level. The photographs were prepared with the same resolution and size. All photographs were displayed for participants on their own devices. It is known that each photograph transmits unique information about the landscape for participants which, in this case, might be used for the online evaluation method [38]. Photographs grouped into their three types are presented in Figure 1.

For evaluation of each landscape photograph, the participants saw photographs on a separate page in the questionnaire. Under each photograph there was the possibility to fill in a Preferred Pleasantness Scale containing three pairs of opposite adjectives: “unpleasant/pleasant”, “I don’t like it/I like it” and “ugly/beautiful”. Pairs were rated on a 7-point Likert scale, from one to seven, with seven indicating the highest level of pleasantness [19]. This scale was correlated with other scales which might measure the restorative quality of the environment and, thus, it helps to predict the usefulness of the landscape for nature-based therapy [39].

### 2.4. Data Analysis

#### 2.4.1. Structural Validity of AEFES

The number of a priori factors in the scale should be confirmed by exploratory factor analysis (EFA) in a validation process [40]. Because the analyzed scale is new, the effects of previous factor analysis are not known.

Two studies were carried out (Study 1: 254 participants, Study 2: 280 participants) to allow the possibility of making comparisons; thus, EFA was applied in each study. Responses to 21 items for both groups were used in a separate analysis, with the maximum likelihood method using direct oblimin rotation (delta = 0) in each case. The decision of the identification of factors in these two studies was based on multiple methods. The factor was classified as importantly occurred if the eigenvalue was higher than 1.0. The scree plot was used for consideration of conceptual meanings of items on each factor. Parallel analysis was also carried out (based on [41]) but, in these analyses, the effect was assessed as too conservative.

#### 2.4.2. Reliability of AEFES

Cronbach’s α [42] reliability coefficient was used to assess the reliability of the subscales of AEFES. Nunally [43] recommended the criteria to evaluate the adequacy of obtained reliability coefficient, with α greater than 0.70. This criterion was used in the current study.

#### 2.4.3. Concurrent, Discriminant and Predictive Validity of AEFES

Concerning concurrent, discriminant and predictive validity, the Pearson correlation between AEFES and the CNS, AmSS-SS and preferred pleasantness measured for each of nine photographs was calculated and analyzed.

## 3. Results

### 3.1. Descriptive and Inferential Statistics

Demographic characteristics for both study groups are included in Table 1. The main difference between Study 1 and Study 2 was that Study 2 involved more young people (55% vs. 35% of participants between 18 and 25 years old). In Study 2, there were also more participants from villages (28.3% in Study 1, 39.60% in Study 2).

The mean values (±SD) for each scale and subscale as well as values for preferred pleasantness for each of nine photographs are shown in Table 2.

For the values of AEFES subscales, the value for “Contact with insects in the forest” was the highest; for “Plants and litter” and “Disgust”, it was moderate, and for “Animals”, it was the lowest. This is connected with distance: animals are far away, whereas insects are very close, on the skin. The mean values of preferred pleasantness were lower for control environments (urban landscape A, B, C), high for forest landscape (D, E, F) and high, but lower than for forest, in the case of a dense forest landscape (G, H and I).

In Study 1, the item-total correlations (each item in subscale correlated with mean values calculated from total items for subscale) for AEFES ranged from 0.707 to 0.789 for “Plants and litter”, from 0.748 to 0.895 for “Animals”, from 0.657 to 0.841 for “Disgust” and from 0.683 to 0.841 for “Insects”. In Study 2, the item-total correlations for AEFES were, respectively, 0.560–0.796, 0.726–0.851, 0.709–0.743 and 0.695–0.853, indicating that good homogeneity occurred in both studies.

### 3.2. Exploratory Factor Analysis (EFA)

For Study 1 (*n* = 254), the Kaiser–Meyer–Olkin (KMO = 0.895) measure of sampling adequacy proves that data from Study 1 were predestined to obtain EFA, and KMO values between 0.8 and 1 indicate that the sampling is adequate (meritorious) [44]. The EFA findings indicated a four-factor solution for the 21 items measured, explaining 61.87% of the total variance in the item scores. All 21 items are appropriately labeled within the four factors as a group of activities inducing discomfort in forest environment: (1) Plants and litter, (2) Animals, (3) Disgust, (4) Insects.

For Study 2 (*n* = 280), the KMO value was = 0.883; thus, these data were adequate for EFA. A four-factor solution was also indicated, explaining 60.14% of the total variance in the item scores. Nineteen items were appropriately loaded within the four factors and labeled as four groups of activities in the forest. Two items had high loadings in many factors and were thus assigned to the same groups as in Study 1 as well as a different factor. The factor pattern coefficients for Study 1 and Study 2 are shown in Table 3.

### 3.3. Reliability of AEFES

The reliability coefficient (Cronbach’s α) was calculated for 21 items for Study 1 (α = 0.910) and Study 2 (α = 0.905). The reliability estimates for Study 1 and Study 2 for the factors “Plants and litter”, “Animals”, “Disgust” and “Insects” are shown in Table 4. Cronbach’s α values for CNS and AmSS-SS are also presented in Table 4. Values of Cronbach’s α obtained in the current research ranged from 0.783 to 0.910 and are seen as high [45,46].

### 3.4. Concurrent Validity

To judge the concurrent validity of the AEFES questionnaire, the relationship between AEFES subscales and AmSS-SS was calculated (Table 4). Subscales of AEFES were positively correlated with values of AmSS-SS. This correlation was not high but was higher in Study 2 than the discriminately validated scale. The most correlated were subscales “Insects” and “Disgust”; the lowest was a correlation with the subscale “Animals”.

### 3.5. Discriminant Validity

To assess the concurrent validity as evidence for the validity of AEFES, the relationship between AEFES and the theoretically suited CNS was calculated. The results of these findings are presented in Table 4. The relationships between these two measures are highly significant or very highly significant or there is no relationship. If a relationship occurs, this will have a negative value. The values of Pearson’s *r* are not high, indicating that these scales are not perfectly correlated, which is expected in the case of discriminant validity. There are differences between studies; in Study 2, the values of Pearson’s *r* are lower.

### 3.6. Predictive Validity

The goal of this research was to judge the possibility of predicting the preferred pleasantness induced by forest landscapes. Thus, the correlations between nine landscape examples were assessed (with three as a control urban landscape). All four subscales of AEFES were not correlated or were slightly positively correlated with the preferred pleasantness of urban landscapes. All subscales were also correlated negatively with preferred pleasantness for all six photographs of a forest landscape. Dense forest landscape was usually connected with slightly higher significant correlation than non-dense forest. The highest correlation is between preferred pleasantness of forest and the “Animals” subscale and the lowest correlation is with the “Insects” subscale. The predictive validity of subscales of AEFES is a fact because the correlations are significant. All correlations are presented in Table 4.

## 4. Discussion

### 4.1. Qualitative Assessment of AEFES

The current study aims to describe a new method of assessing the preferred pleasantness induced by urban and forest environments and to validate the instrument developed for this purpose: the AEFES. It is worth noting that preferred pleasantness is a construct that consists of many dimensions and the AEFES scale captures only some of them. The four-factor structure of the questionnaire was confirmed in an exploratory factor analysis using excluded factors that suggest the vulnerability of self-boundaries of subjects for contact with plants and litter in the forest, animals in the forest, disgust in the forest and insects in the forest. The subscales used have high reliability (αs = 0.783 to 0.859). This relevance was confirmed in two independent online studies.

As theoretically expected, the instrument showed that four subscales were positively correlated to the Amoebic Self Scale’s spatial–symbolic domain (concurrent validity) and negatively correlated with the CNS (discriminant validity), and three of the four subscales were correlated negatively with preferred pleasantness (predictive validity). This suggests that AEFES might have the potential to measure real phenomena, as it is connected with the preferred pleasantness induced by forest environments. To sum up, the AEFES is a reliable and valid instrument with practical use for measuring an anti-environmental forest experience, which might be useful for the prediction of the preferred pleasantness of subjects towards forest environments.

### 4.2. Theoretical Integration

It is worth mentioning that there is probably some psychological mechanism which, by inducing fear or disgust, divides the body of a subject from the natural environment in the forest. Contact in these other situations might be harmful or dangerous to the health of a subject’s body, so fear or disgust responses protect the body before it comes into contact with a potentially dangerous environment. The “Plants or litter” subscale of AEFES provides information about the vulnerability of a subject’s hypothetical self for contact with trees, other plants and litter in the forest environment. The mean values of this scale in both analyzed studies were moderate, which indicates that this environmental feature might have a moderate level in the analyzed samples. The subscale “Animals” had the lowest mean values in both samples (in comparison to other subscales); thus, components of the forest environment like large herbivores or small mammals, which are not dangerous, are not seen as harmful from the perspective of self-boundaries. This subscale is also slightly correlated with AmSS-SS, the slightest values from all subscales of AEFES, but this subscale has good predictive validity and might also be used for prediction of the level of preferred pleasantness of subject. The subscale “Disgust” had moderate values and was significantly correlated with AmSS-SS. This subscale measures self-boundaries which are vulnerable for interaction with disgust or undesirable items, which might be seen, touched or smelled in the forest. This subscale has good predictive validity. The last subscale, “Insects”, contains items which describe the interaction with insects in the forest and also concerns other organisms like ticks or spiders (from other groups of animals). This scale had the highest mean values but has the least predictive validity. This means that subjects observing photographs of forest environments have the worst ability to predict the occurrence of lower values of preferred pleasantness but can have some negative attitude against “Insects”.

This finding supports both theories included in the introduction: if the object is far from a subject’s skin, it is probably far from the self-boundaries, so it is connected with Amoebic Self Theory; if the object is far from a subject’s skin, there is some prospect (distance between subject and object), showing that the Prospect and Refuge Theory may be relevant. That these two theories address and allow measurement of the same things was only possible to ascertain after this research and discussion.

### 4.3. Limitations

As mentioned in the introduction, other questionnaires cannot be used in these studies for various reasons. The Subiza-Pérez [47] study used the Natural Environment Scoring Tool and the Place Attachment and Place Identification Scale [48] questionnaire to predict the restorative quality of the environment. However, these questionnaires were not used in relation to the forest environment of the temperate zone and they did not mention an important aspect—the proximity to the body of the examined objects. Therefore, the analyzed AEFES scale is much more comprehensive in this respect. The aforementioned research also does not address issues related to the different perceptions of forest landscapes resulting from the density of vegetation and therefore the possibility of hiding insects and potential predators. In the current research, it was proven that dense forest is less pleasant for the respondents, which explains, among other things, the observed correlation between preferred pleasantness and the values of the “Insects” subscale.

Presumably, AEFES can also be used to predict psychological relaxation. This prediction can be made by qualified personnel who would like to determine whether a hypothetical participant of forest recreation can benefit from this activity in the form of psychological relaxation, i.e., improved mood, an increased level of positive emotions, a decreased level of negative emotions and regeneration of mental strength (increased restorativeness). Other studies have predicted the level of psychological relaxation of the natural environment using scales measuring place attachment and place identification [47], so the AEFES scale will also probably be a scale that can be used for this purpose, but this requires further research.

Since preferred pleasure is correlated with restorativeness [19] and restorativeness is related to the positive properties caused by the forest, among other things, in the examined patients of a psychiatric hospital [48], it can be concluded that the subjects experiencing the pleasure of viewing pleasant pictures of the forest landscape will experience beneficial effects on their health. However, confirmation of this fact requires future studies in order to verify whether the AEFES scale may be useful in predicting the therapeutic effect for patients. The AEFES questionnaire can also be used to predict the benefits that participants of forest therapy may derive in various areas that may be affected by the forest: psychological effects, physiological effects and effects on social wellbeing. In addition, different features of the forest may have a different impact on the respondents (different forms of forest use, different species or different types of forests, including those resulting from geographic diversity), which can be predicted using the AEFES scale [49,50].

The limitation of this study may be the use of virtual examples of the forest environment in the form of photos, instead of landscape in a natural environment. However, looking at a virtual forest landscape is also associated with many benefits that can be derived by the respondents. A good example is a study in which people looking at films from forest areas experienced relaxation, but this also decreased their willingness to procrastinate [51]. In other studies, looking at a virtual forest reduced stress in adults [52], and the respondents’ relationships to the virtual forest environment were varied [53].

It is also worth noting that different environments will not necessarily be suitable for forest therapy, which is an implication of the Prospects and Refuges Theory [22]. On the other hand, the diversity of the environments in which the participants of forest therapy stay is of great importance for the therapy, where the diversity of the environment and the presence of open space is also important [54,55].

The AEFES questionnaires are not the only tool to predict the expected benefits that contact with nature may bring to its participants. Subjects with acute depressive symptoms preferred dark and dense forest landscapes [56]; experts and laypeople might have different perceptions of tree features [10]. In addition, individual preferences were important in the mental and physical reactions of the respondents to the forest environment [9].

Future research should be carried out to check whether, for example, the statement “feel one with nature” included in the CNS scale will be related to receiving psychological benefits related to nature. The analysis of the available literature shows that this relationship has not yet been studied.

It is possible that not all of the statements in the AEFES scale will always reflect real fear in the forest. For example, a person who claims to experience discomfort in the forest as a result of contact with a snake may not feel this discomfort in the real environment, and vice versa. This requires empirical testing in future research. In these studies, however, an image of the situation in the forest was presented, and it is highly likely that this image of discomfort is real, as evidenced by different responses to different landscapes viewed.

On this scale, items about discomfort that could be caused by deadwood were not included. Neither of the items concerned this. It is planned in future studies to supplement the AEFES scale with statements regarding possible discomfort resulting from the perception of deadwood in the forest.

In these studies, half of the respondents were people using forest areas regularly and half of the respondents did not use forest areas (in both groups). Therefore, some of the respondents are representative people using the forest and some are not. This diversification was intended to ensure that all groups are considered equally, which makes the results plausible—as they show the distribution of actual use of the forest environment by social media society. Additionally, the respondents presented different levels of education, which was also intended to reflect the society using social media, which could potentially benefit from computer-mediated forest therapy in the future.

### 4.4. Implications and Future Research

The ability to predict the restorativeness of the environment (which is correlated with pleasantness [57]) is used in a small number of scientific articles regarding the possibility to predict pleasantness or restorativeness [19,58]. This study is innovative in the creation of a novel instrument designed exactly for the prediction of benefits taken from natural forest environment. The study showed that it was possible to predict social media users’ possibility to like or evaluate positively each forest environment. The AEFES also has potential to be used by physicians or therapists working with depression or anxiety because forest environments might be an additional remedy for those patients with psychological problems [48], and prediction of pleasantness felt by each patient from described contact with forest environment is possible with the use of this instrument.

Future research is needed concerning AEFES. In the current study, only the prediction of preferred pleasantness was tested. Because other measurements of the impact of forest or natural environments are also possible, it is worth considering the verification of the predictive potential of AEFES for this measure. Restorativeness is one possible measure of effects. The current study also only verified the photographic illustration of the forest environment influence in relation to AEFES vs. preferred pleasantness. In future studies, it will be very important to use the proposed instrument to predict effects in real forest environments.

Moreover, in future research, the usage of confirmatory factor analysis (CFA) for analysis should be considered. This research planned and performed comparisons of two samples for EFA, but because CFA cannot be done on the same sample as EFA, CFA could not be done in this research.

The current problems with the COVID-19 pandemic [59] also bring new possibilities for environmental studies on the influence of virtual forest environments on humans. Many people are living in isolation during the pandemic, sometimes with no contact with the outside environment. Some form of replacement of interaction with these outside environments, such as photographs of a forest environment, might be needed because of the natural need of people to be in contact with nature [60]. Thus, the instrument is needed to personalize these photographs or films to increase the pleasantness experienced by the subjects. It can be imagined that a tree stand (landscape) with different characteristics may be better suited to the needs of the person who would benefit from viewing it. For example, a given person gains the benefits of viewing both the stand and the dense stand (which was predicted by the AEFES scale); therefore, for such a person, there are no contraindications for practicing forest therapy in both stands.

## 5. Conclusions

In the current study, the new questionnaire designed for the prediction of preferred pleasantness induced by photographs of forest environments was proposed and psychometrically validated. AEFES was demonstrated to be reliable and valid. Factor analysis confirmed that the four-factor structure is relevant; thus, four subscales were proposed: “Plants and litter”, “Animals”, “Disgust” and “Insects”, with each subscale relevant to one of four groups of experiences which might occur in the temperate forest environment. Two theories were also discussed and tested for their applicability for use in explaining the results of prediction. Further studies are needed to test the psychometric predictive validity of the instrument for restorativeness of virtual and natural environments, and usability in the COVID-19 era should be considered.

It is worth summarizing, however, that the AEFES scale has some limitations mentioned in this manuscript that should be investigated in future experiments. This scale, however, may explain some of the dependencies that account for the preferred pleasure experienced by people viewing pictures of the forest, videos of the forest or finally the forest itself. This relationship needed to be investigated and the results show that it is a significant relationship.

## Figures and Tables

**Table 1:** Demographic characteristics of two groups of participants (Study 1 and Study 2).

Demographic Characteristic	Study 1			Study 2		
	Frequency	Percent	Cumulative Percent	Frequency	Percent	Cumulative Percent
Gender						
Male	79	31.10	31,10	113	40.40	40.40
Female	175	68.90	10,000	167	59.60	100.00
Age (groups)						
18–25 years	89	35.04	35.04	154	55.00	55.00
26–40 years	77	30.03	65.07	88	31.00	86.00
41 or more years	88	34.64	100.00	38	14.00	100.00
Place of living						
City > 250 thousand	63	24.80	71.70	51	18.20	60.40
City < 250 thousand	36	14.20	21.70	24	8.60	19.30
City < 100 thousand	19	7.50	7.50	30	10.70	10.70
City < 50 thousand	64	25.20	46.90	64	22.90	42.10
Village	72	28.30	100.00	111	39.60	100.00
Educational background						
Primary school	4	1.60	1.60	7	2.50	2.50
High school	130	51.20	52.80	130	46.40	48.90
Higher education (bachelor)	50	19.70	72.40	69	24.60	73.60
Higher education (more than bachelor)	70	27.60	100.00	74	26.40	100.00
Material standing (self-evaluation)						
Very bad	1	0.40	8.30	3	1.10	1.10
Bad	5	2.00	8.70	6	2.10	3.20
Medium	130	51.20	46.90	114	40.70	43.90
Good	97	38.20	98.00	129	46.10	90.00
Very good	21	8.30	100.00	28	10.00	100.00
Activity						
Work and study	27	10.63	10.63	39	13.93	13.93
Study	49	19.29	29.92	79	28.21	42.14
Work	166	65.35	95.28	150	53.57	95.71
Unemployed	12	4.72	100.00	12	4.29	100.00

**Table 2:** Means and standard deviations of each scale and subscale and mean preferred pleasantness for each photograph.

Name of Variable	Study 1		Study 2	
	Mean	SD	Mean	SD
Plants and litter	2.36	1.14	2.23	1.09
Animals	1.56	0.95	1.62	1.02
Disgust	3.26	1.51	3.55	1.56
Insects	4.76	1.48	4.72	1.46
CNS	5.08	1.03	5.35	1.09
AmSS-SS	-	-	3.98	1.26
Urban landscape (A)	-	-	1.85	1.00
Urban landscape (B)	-	-	1.82	0.95
Urban landscape (C)	-	-	2.34	1.38
Forest landscape (D)	-	-	6.20	1.26
Forest landscape (E)	-	-	5.87	1.37
Forest landscape (F)	-	-	5.65	1.48
Dense forest landscape (G)	-	-	5.52	1.45
Dense forest landscape (H)	-	-	5.23	1.61
Dense forest landscape (I)	-	-	5.26	1.63
Urban landscape	-	-	2.00	0.91
Forest landscape	-	-	5.91	1.23
Dense forest landscape	-	-	5.34	1.41

**Table 3:** Rotated factor pattern matrix for the Anti-Environmental Forest Experience Scale (AEFES).

Item Number	Item	Plants and Litter	Animals	Disgust	Insects
	Study 1				
1	Head blow by a falling cone	**0.768**	−0.021	0.097	0.14
2	Being hit by a tree branch	**0.643**	−0.024	0.012	−0.133
3	Stepping into mud	**0.592**	−0.122	0.068	−0.157
4	Breaking through the thickets	**0.587**	−0.151	−0.016	−0.154
5	Collision with a tree	**0.459**	−0.006	0.197	−0.047
6	Smell of rotting plant matter	**0.388**	−0.37	0.087	−0.07
7	Seeing a deer with antlers from afar	−0.084	**−0.973**	−0.04	−0.034
8	Seeing a fox from afar	−0.025	**−0.73**	0.209	−0.003
9	Seeing deer from afar	0.184	**−0.724**	−0.132	0.12
10	Seeing a running mouse	−0.009	**−0.492**	0.184	−0.032
11	Seeing a slithering snake	−0.038	−0.158	**0.756**	−0.023
12	Noticing a dead animal	0.017	0.003	**0.585**	0.051
13	Walking on unstable ground in the forest	0.252	0.053	**0.553**	−0.068
14	Mouse/mice crawling on your body	0.051	−0.018	**0.543**	−0.219
15	Seeing a boar from afar	0.044	−0.119	**0.378**	−0.142
16	Being bitten by a “horse fly”	0.225	0.059	−0.027	**−0.728**
17	Being attacked by insects	0.301	0.021	−0.062	**−0.727**
18	Infection from a tick bite	−0.2	0.037	0.092	**−0.63**
19	Tick bite	−0.057	−0.111	0.17	**−0.619**
20	Ants crawling on your body	0.268	0.028	0.165	**−0.53**
21	Spider web sticking	0.276	−0.157	−0.017	**−0.389**
	Study 2				
1	Head blow by a falling cone	**0.744**	0.032	0.088	−0.03
2	Being hit by a tree branch	**0.512**	−0.033	0.032	0.22
3	Stepping into mud	**0.81**	−0.033	−0.215	−0.092
4	Breaking through the thickets	**0.587**	0.053	−0.08	0.097
5	Collision with a tree	**0.565**	−0.003	0.023	0.129
6	Smell of rotting plant matter	**0.272**	0.358	−0.22	−0.001
7	Seeing a deer with antlers from afar	−0.037	**0.812**	−0.066	0.08
8	Seeing a fox from afar	−0.07	**0.669**	−0.147	0.078
9	Seeing deer from afar	0.015	**0.902**	0.224	−0.089
10	Seeing a running mouse	0.027	**0.466**	−0.17	−0.047
11	Seeing a slithering snake	−0.032	0.102	**−0.61**	0.102
12	Noticing a dead animal	0.062	0.173	**−0.33**	0.336
13	Walking on unstable ground in the forest	0.347	0.018	**−0.586**	−0.049
14	Mouse/mice crawling on your body	−0.024	0.117	**−0.382**	0.308
15	Seeing a boar from afar	0.118	0.264	**−0.354**	0.154
16	Being bitten by a “horse fly”	0.15	−0.014	0.115	**0.768**
17	Being attacked by insects	0.13	0.03	0.054	**0.782**
18	Infections from a tick bite	−0.165	−0.116	−0.112	**0.753**
19	Tick bite	0.027	0.093	−0.036	**0.65**
20	Ants crawling on your body	0.222	−0.013	−0.101	**0.557**
21	Spider web sticking	0.25	0.114	−0.034	**0.39**

Bold factors build one specific factor in the table.

**Table 4:** Correlations and internal consistency of AEFES, CNS, AmSS-SS and preferred pleasantness induced by photographs of different landscapes for Study 1 and Study 2 (*n* = 254 and *n* = 280).

Connectedness to Nature, Spatial–Symbolic Aspect of AmSS and Pleasantness of Photographs of Different Landscapes	Plants and Litter	Animals	Disgust	Insects
	Study 1 (α = 0.841); Study 2 (α = 0.809)	Study 1 (α = 0.836); Study 2 (α = 0.795)	Study 1 (α = 0.783); Study 2 (α = 0.788)	Study 1 (α = 0.852); Study 2 (α = 0.859)
Study 1				
CNS (α = 0.879)	−0.335 **	−0.389 **	−0.284 **	−0.192 **
Study 2				
CNS (α = 0.860)	−0.289 ***	−0.182 **	−0.198 **	−0.077
AmSS-SS (α = 0.778)	0.265 ***	0.176 **	0.300 **	0.310 ***
Urban landscape (A)	0.101	0.04	0.027	0.035
Urban landscape (B)	0.11	−0.012	−0.01	0.047
Urban landscape (C)	0.153 *	0.122 *	0.122 *	0.109
Forest landscape (D)	−0.051	−0.098	0.022	0.098
Forest landscape (E)	−0.163 **	−0.169 **	−0.09	−0.042
Forest landscape (F)	−0.331 ***	−0.300 ***	−0.282 ***	−0.192 **
Dense forest landscape (G)	−0.151 *	−0.124 *	−0.063	−0.026
Dense forest landscape (H)	−0.227 ***	−0.292 ***	−0.263 ***	−0.150 *
Dense forest landscape (I)	−0.279 ***	−0.277 ***	−0.276 ***	−0.176 **
Urban landscape	0.152 *	0.072	0.068	0.084
Forest landscape	−0.210 ***	−0.216 ***	−0.139 *	−0.059
Dense forest landscape	−0.246 ***	−0.261 ***	−0.229 ***	−0.134 *

*** Correlation is significant at the 0.001 level (2-tailed). ** Correlation is significant at the 0.01 level (2-tailed). * Correlation is significant at the 0.05 level (2-tailed).
